# Supplementary material for: Insights into the Microbiome and Antibiotic Resistance Genes from Hospital Environmental Surfaces: A Prime Source of Antimicrobial Resistance
Source: Antibiotics (Basel). 2024 Jan 26;13(2):127. doi: 10.3390/antibiotics13020127 (PMC10885873; doi:10.3390/antibiotics13020127)
Supplement: Supplementary file 1 [file antibiotics-13-00127-s001.zip › antibiotics-2811109-supplementary.pdf]

Figure S1: Rarefaction analysis of samples

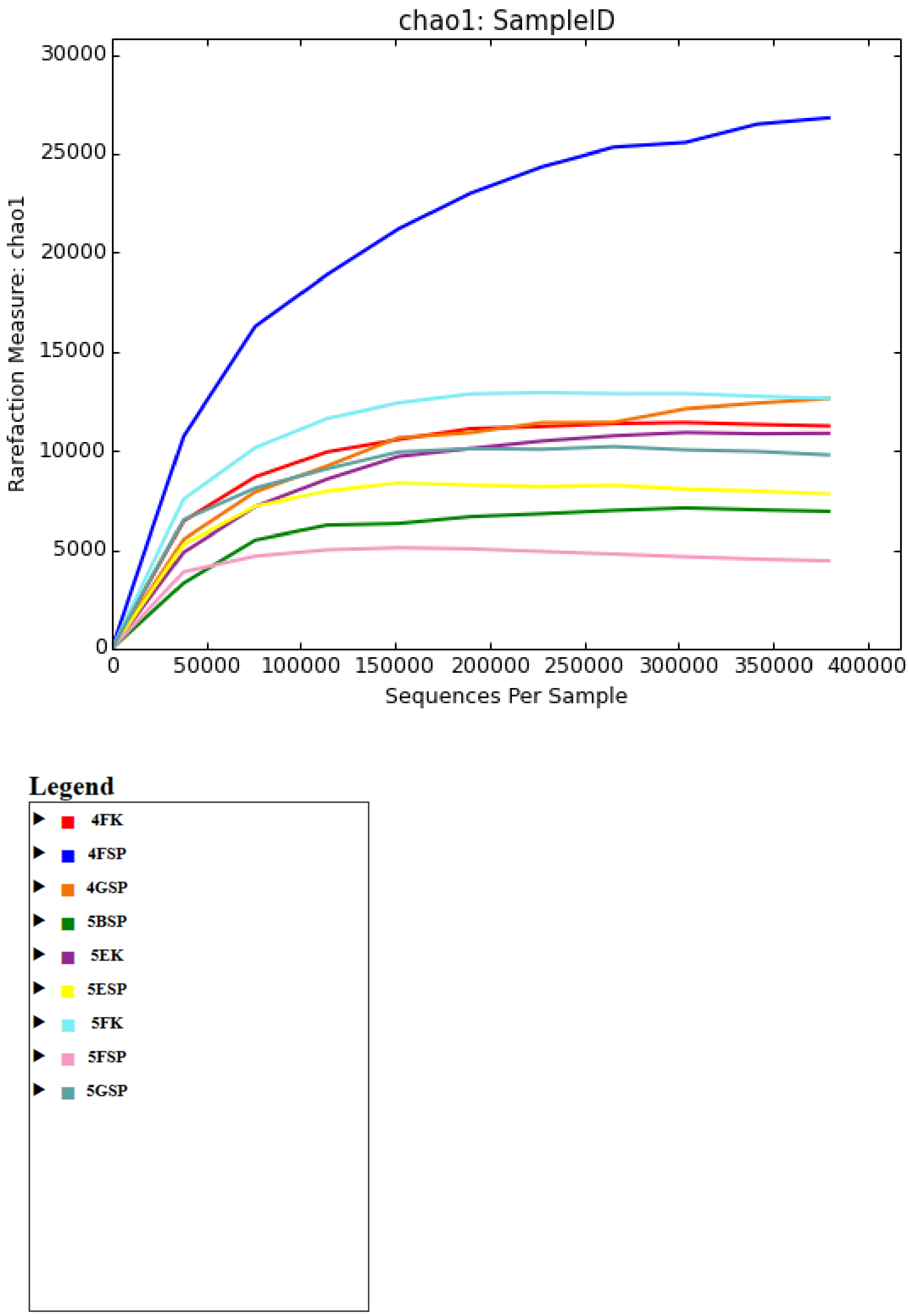

Figure S2: Jackknifed unweighted pair group method with arithmetic mean (UPGMA) analysis of sample relatedness

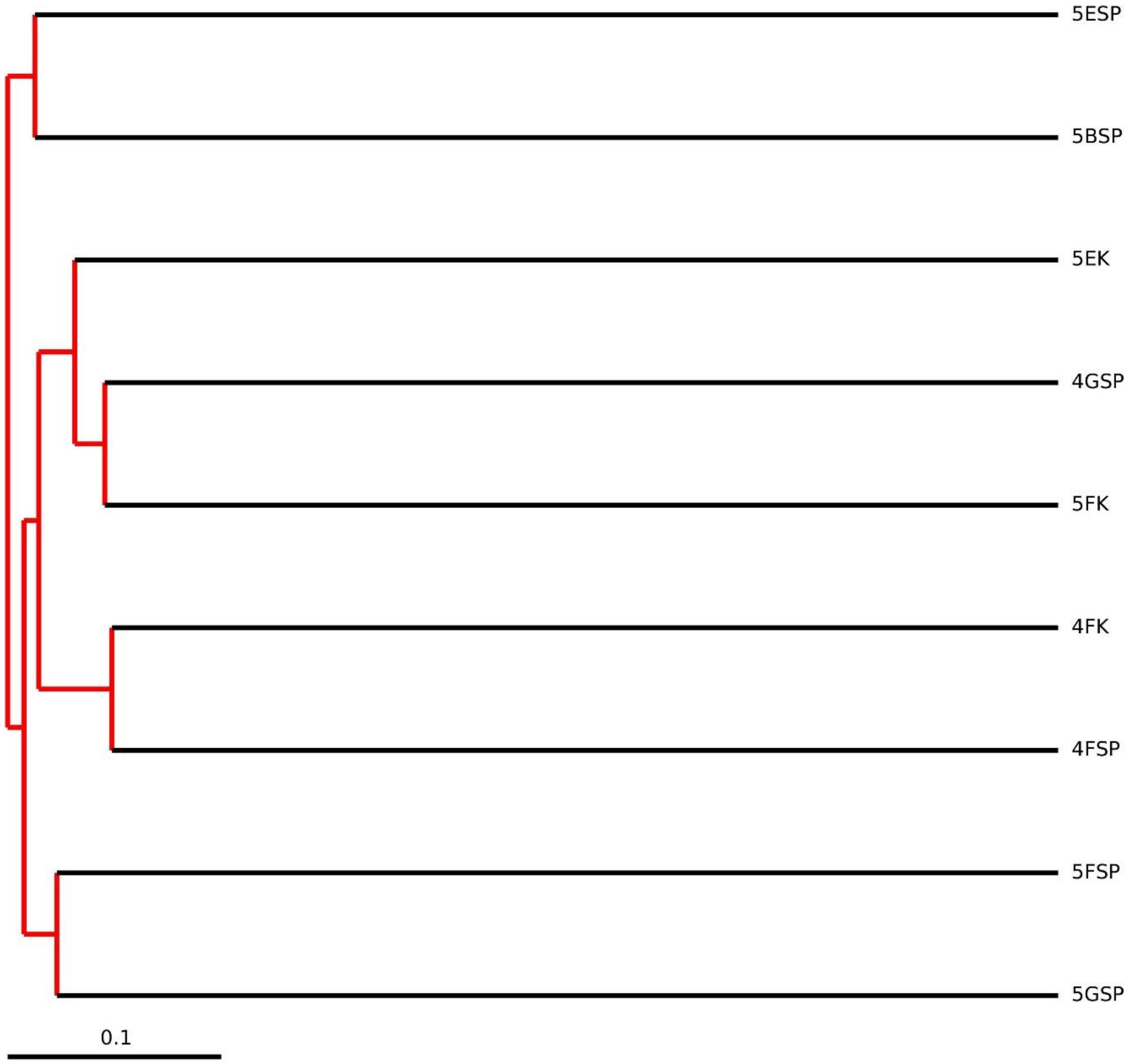

Table S1: Sequencing Output of each selected sample generated from the NovaSEQ6000 run. The total output is the summation of total bases from read 1 and read 2. The total base in each read is the multiplication of the number of reads with 150 bp (the read length configuration of NovaSEQ6000). E.g. 1,000 reads = 1,000 x 150 bp = 150,000 bp.

file	# reads	# bases	min_len
4FK_DKDN220007726-1A_HVJLYDSX3_L2_1.fq.gz	27,218,837	4,082,825,550	150
4FK_DKDN220007726-1A_HVJLYDSX3_L2_2.fq.gz	27,218,837	4,082,825,550	150
4FSP_DKDN220007725-1A_HVJLYDSX3_L2_1.fq.gz	29,105,407	4,365,811,050	150
4FSP_DKDN220007725-1A_HVJLYDSX3_L2_2.fq.gz	29,105,407	4,365,811,050	150
4GSP_DKDN220007727-1A_HVJLYDSX3_L2_1.fq.gz	25,367,995	3,805,199,250	150
4GSP_DKDN220007727-1A_HVJLYDSX3_L2_2.fq.gz	25,367,995	3,805,199,250	150
5BSP_DKDN220007733-1A_HVJLYDSX3_L2_1.fq.gz	30,284,713	4,542,706,950	150
5BSP_DKDN220007733-1A_HVJLYDSX3_L2_2.fq.gz	30,284,713	4,542,706,950	150
5EK_DKDN220007732-1A_HVJLYDSX3_L2_1.fq.gz	31,277,941	4,691,691,150	150
5EK_DKDN220007732-1A_HVJLYDSX3_L2_2.fq.gz	31,277,941	4,691,691,150	150
5ESP_DKDN220007731-1A_HVJLYDSX3_L2_1.fq.gz	31,559,160	4,733,874,000	150
5ESP_DKDN220007731-1A_HVJLYDSX3_L2_2.fq.gz	31,559,160	4,733,874,000	150
5FK_DKDN220007730-1A_HVJLYDSX3_L2_1.fq.gz	31,333,139	4,699,970,850	150
5FK_DKDN220007730-1A_HVJLYDSX3_L2_2.fq.gz	31,333,139	4,699,970,850	150
5FSP_DKDN220007729-1A_HVJLYDSX3_L2_1.fq.gz	34,715,441	5,207,316,150	150
5FSP_DKDN220007729-1A_HVJLYDSX3_L2_2.fq.gz	34,715,441	5,207,316,150	150
5GSP_DKDN220007728-1A_HVJLYDSX3_L2_1.fq.gz	32,388,476	4,858,271,400	150
5GSP_DKDN220007728-1A_HVJLYDSX3_L2_2.fq.gz	32,388,476	4,858,271,400	150

Table S2: Total number of reads and lengths in each sample, filtered based on contig lengths. All statistics are based on contigs of size >= 500 bp, unless otherwise noted (e.g., "# contigs (>= 0 bp)" and "Total length (>= 0 bp)" include all contigs).

Assembly	4FK	4FSP	4GSP	5BSP	5EK	5ESP	5FK	5FSP	5GSP
# contigs (>= 0 bp)	2,380	8,672	3,836	11,990	7,018	76,931	22,205	31,236	7,626
# contigs (>= 1000 bp)	638	4,284	971	1,599	612	20,619	3,187	8,316	2,687
# contigs (>= 5000 bp)	260	1,048	168	429	199	4,176	266	2,168	563
# contigs (>= 10000 bp)	190	552	99	249	159	1,785	131	1,155	292
# contigs (>= 25000 bp)	119	300	69	81	106	377	48	406	146
# contigs (>= 50000 bp)	74	196	45	41	69	52	18	126	85
Total length (>= 0 bp)	12,540,520	45,631,337	11,089,460	17,612,687	17,532,611	111,615,183	19,571,929	61,476,756	24,365,502
Total length (>= 1000 bp)	11,762,496	43,132,447	9,694,650	12,446,635	14,414,525	85,922,540	11,350,067	51,308,981	21,960,288
Total length (>= 5000 bp)	10,939,114	36,212,965	8,050,189	10,485,494	13,787,427	52,219,069	6,328,746	38,323,345	17,370,591
Total length (>= 10000 bp)	10,423,870	32,780,160	7,596,935	9,185,377	13,503,651	35,550,213	5,386,923	31,196,384	15,500,710
Total length (>= 25000 bp)	9,268,569	29,006,339	7,091,885	6,508,673	12,598,940	14,454,400	4,143,152	19,539,962	13,265,122
Total length (>= 50000 bp)	7,586,459	25,198,007	6,206,804	5,148,701	11,208,732	3,598,331	3,153,050	9,690,222	11,160,829
Assembly	4FK	4FSP	4GSP	5BSP	5EK	5ESP	5FK	5FSP	5GSP
# contigs	1,135	6,825	2,030	5,710	3,019	39,555	8,786	15,251	4,441

Largest contig	326,082	767,790	381,642	288,066	690,339	148,179	868,576	208,852	994,937
Total length	12,093,482	44,944,561	10,407,429	15,198,379	16,021,175	99,107,123	15,210,266	56,099,031	23,181,746
GC (%)	40.50	53.50	32.89	58.28	36.23	54.18	48.68	51.94	52.01
N50	64,619	67,939	84,935	18,385	126,407	5,640	2,655	13,174	42,331
N75	29,191	8,169	6,512	1,715	38,292	1,785	991	3,320	4,984
L50	48	149	30	133	30	3,675	637	881	95
L75	112	655	126	759	88	11,850	3,245	3,093	567
# N's per 100 kbp	-	-	-	-	-	-	-	-	-

Table S3: Identification of AMR genes from *de novo* assembly [NCBI AMRPlusFinder database]

#FILE	SEQUENCE	GENE	%COVERAGE	%IDENTITY	DATABASE	ACCESSION	PRODUCT	RESISTANCE

4FK.fna	4FK_k141_1537_65	blaOXA-699	100	99.88	ncbi	NG_062321.1	OXA-51 family carbapenem-hydrolyzing class Dbeta-lactamase OXA-699	BETA-LACTAM
4FK.fna	4FK_k141_163_11	ant(3")-IIa	100	99.11	ncbi	NG_054648.1	aminoglycoside nucleotidyltransferase ANT(3")-IIa	SPECTINOMYCIN; STREPTOMYCIN
4FK.fna	4FK_k141_1972_2	blaZ	100	100	ncbi	NG_047532.1	penicillin-hydrolyzing class Abeta-lactamase BlaZ	BETA-LACTAM
4FK.fna	4FK_k141_1972_3	blaR1-2	100	100	ncbi	NG_047537.1	beta-lactam sensor/signal transducer BlaR1	BETA-LACTAM
4FK.fna	4FK_k141_1972_4	blaI_of_Z	100	99.74	ncbi	NG_047499.1	penicillinase repressor BlaI	BETA-LACTAM
4FK.fna	4FK_k141_1988_6	mecA	100	99.95	ncbi	NG_047940.1	PBP2a family beta-lactam-resistantpeptidoglycan transpeptidase MecA	METHICILLIN
4FK.fna	4FK_k141_2082_30	blaADC-32	100	100	ncbi	NG_050717.1	class C extended-spectrumbeta-lactamase ADC-32	CEPHALOSPORIN
4FK.fna	4FK_k141_2181_166	dfrC	100	100	ncbi	NG_047752.1	trimethoprim-resistant dihydrofolatereductase DfrC	TRIMETHOPRIM
4FK.fna	4FK_k141_2324_4	msr(A)	100	100	ncbi	NG_048001.1	ABC-F type ribosomal protectionprotein Msr(A)	MACROLIDE
4FK.fna	4FK_k141_2378_49	fosB-251804940	100	99.77	ncbi	NG_047888.1	FosB family fosfomycin resistancebacillithiol transferase	FOSFOMYCIN
4FSP.fna	4FSP_k141_1630_15	aph(3')-IIb	100	99.13	ncbi	NG_047424.1	aminoglycoside O-phosphotransferase APH(3')-IIb	KANAMYCIN
4FSP.fna	4FSP_k141_1630_24	blaPDC-216	100	99.92	ncbi	NG_055279.1	class C beta-lactamase PDC-216	CEPHALOSPORIN
4FSP.fna	4FSP_k141_2377_132	fosA6	100	99.29	ncbi	NG_051497.1	fosfomycin resistance glutathionetransferase FosA6	FOSFOMYCIN
4FSP.fna	4FSP_k141_2418_339	oqxB25	100	99.91	ncbi	NG_050444.1	multidrug efflux RND transporterpermease subunit OqxB25	PHENICOL;QUINOLONE
4FSP.fna	4FSP_k141_2418_340	oqxA10	100	99.58	ncbi	NG_050418.1	multidrug efflux RND transporter periplasmic adaptor subunit OqxA10	PHENICOL;QUINOLONE
4FSP.fna	4FSP_k141_374_3	fosA_gen	61.43	93.02	ncbi	NG_047881.1	FosA family fosfomycin resistanceglutathione transferase	FOSFOMYCIN
4FSP.fna	4FSP_k141_3814_2	blaACT-28	100	100	ncbi	NG_048614.1	cephalosporin-hydrolyzing class Cbeta-lactamase ACT-28	CEPHALOSPORIN
4FSP.fna	4FSP_k141_4775_29	blaADC-156	100	100	ncbi	NG_055286.1	class C beta-lactamase ADC-156	CEPHALOSPORIN
4FSP.fna	4FSP_k141_4825_24	blaACT-25	100	99.65	ncbi	NG_048612.1	cephalosporin-hydrolyzing class Cbeta-lactamase ACT-25	CEPHALOSPORIN
4FSP.fna	4FSP_k141_4912_98	blaOXA-395	100	99.87	ncbi	NG_049684.1	OXA-50 family oxacillin-hydrolyzingclass D beta-lactamase OXA-395	BETA-LACTAM
4FSP.fna	4FSP_k141_5059_11	ant(3")-IIa	100	99.37	ncbi	NG_054646.1	aminoglycoside nucleotidyltransferase	SPECTINOMYCIN; STREPTOMYCIN

							ANT(3")-IIa	
4FSP.fna	4FSP_k141_5616_2	mcr-10.1	100	100	ncbi	NG_066767.1	phosphoethanolamine--lipid A transferase MCR-10.1	COLISTIN
4FSP.fna	4FSP_k141_6064_18	fosB-38141535	100	98.8	ncbi	NG_047887.1	FosB family fosfomycin resistance bacillithiol transferase	FOSFOMYCIN
4FSP.fna	4FSP_k141_6324_2	fosA	100	96.01	ncbi	NG_050405.1	fosfomycin resistance glutathione transferase FosA	FOSFOMYCIN
4FSP.fna	4FSP_k141_650_39	sul1	100	100	ncbi	NG_048082.1	sulfonamide-resistant dihydropteroate synthase Sul1	SULFONAMIDE
4FSP.fna	4FSP_k141_650_41	aadA2	98.48	100	ncbi	NG_051846.1	ANT(3")-Ia family aminoglycoside nucleotidyltransferase AadA2	STREPTOMYCIN
4FSP.fna	4FSP_k141_650_42	dfrA15	100	100	ncbi	NG_047702.1	trimethoprim-resistant dihydrofolate reductase DfrA15	TRIMETHOPRIM
4FSP.fna	4FSP_k141_650_66	qnrS1	100	100	ncbi	NG_050543.1	quinolone resistance pentapeptide repeat protein QnrS1	QUINOLONE
4FSP.fna	4FSP_k141_650_70	blaLAP-2	100	100	ncbi	NG_049264.1	class A beta-lactamase LAP-2	BETA-LACTAM
4FSP.fna	4FSP_k141_6847_10	blaOXA-699	100	99.88	ncbi	NG_062321.1	OXA-51 family carbapenem-hydrolyzing class D beta-lactamase OXA-699	BETA-LACTAM
4FSP.fna	4FSP_k141_6853_346	bla1	99.46	96	ncbi	NG_055637.1	class A beta-lactamase Bla1	BETA-LACTAM
4FSP.fna	4FSP_k141_6912_119	blaSHV-27	100	100	ncbi	NG_050064.1	class A extended-spectrum beta-lactamase SHV-27	CEPHALOSPORIN
4FSP.fna	4FSP_k141_7095_69	blaCSA-1	100	99.03	ncbi	NG_062223.1	class C beta-lactamase CSA-1	CEPHALOTHIN
4FSP.fna	4FSP_k141_7185_44	satA_Ba	98.74	91.34	ncbi	NG_064661.1	streptothricin N-acetyltransferase SatA	STREPTOTHRICIN
4FSP.fna	4FSP_k141_7324_1	oqxA3	52.3	92.85	ncbi	NG_050421.1	multidrug efflux RND transporter periplasmic adaptor subunit OqxA3	PHENICOL;QUINOLON E
4FSP.fn	4FSP_k141_		100	99.51	ncbi	NG_047883.1		FOSFOMYCIN

a	7576_155	fosA-354 827590					FosA family fosfomycin resistance glutathione transferase	
4FSP.fna	4FSP_k141_7886_12	BcII	100	99.87	ncbi	NG_055630.1	BcII family subclass B1 metallo-beta-lactamase	CARBAPENEM
4FSP.fna	4FSP_k141_8463_244	catB7	100	99.37	ncbi	NG_047614.1	type B-4 chloramphenicol O-acetyltransferase CatB7	CHLORAMPHENICOL
4FSP.fna	4FSP_k141_8896_6	tet(C)	100	100	ncbi	NG_048181.1	tetracycline efflux MFS transporter Tet(C)	TETRACYCLINE
4GSP.fna	4GSP_k141_1198_3	blaZ	100	96.34	ncbi	NG_055999.1	penicillin-hydrolyzing class A beta-lactamase BlaZ	BETA-LACTAM
4GSP.fna	4GSP_k141_1198_4	blaR1	100	92.78	ncbi	NG_047539.1	beta-lactam sensor/signal transducer BlaR1	BETA-LACTAM
4GSP.fna	4GSP_k141_1198_5	blaI_of_Z	100	95.54	ncbi	NG_047499.1	penicillinase repressor BlaI	BETA-LACTAM
4GSP.fna	4GSP_k141_1256_1	dfrG	100	100	ncbi	NG_047756.1	trimethoprim-resistant dihydrofolate reductase DfrG	TRIMETHOPRIM
4GSP.fna	4GSP_k141_2448_2	msr(A)	100	99.93	ncbi	NG_055998.1	ABC-F type ribosomal protection protein Msr(A)	MACROLIDE
4GSP.fna	4GSP_k141_2448_3	mph(C)	100	100	ncbi	NG_047991.1	Mph(C) family macrolide 2'-phosphotransferase	MACROLIDE
4GSP.fna	4GSP_k141_284_7	fusB	100	100	ncbi	NG_047900.1	fusidic acid resistance EF-G-binding protein FusB	FUSIDIC_ACID
4GSP.fna	4GSP_k141_3073_108	dfrC	100	96.71	ncbi	NG_047752.1	trimethoprim-resistant dihydrofolate reductase DfrC	TRIMETHOPRIM
4GSP.fna	4GSP_k141_309_60	fosB-251 804940	100	97.2	ncbi	NG_047889.1	FosB family fosfomycin resistance bacillithiol transferase	FOSFOMYCIN
4GSP.fna	4GSP_k141_3848_58	tet(38)	99.78	91.78	ncbi	NG_055983.1	tetracycline efflux MFS transporter Tet(38)	TETRACYCLINE
4GSP.fna	4GSP_k141_585_1	vga(A)-LC	100	100	ncbi	NG_047097.1	ABC-F type ribosomal protection protein Vga(A)-LC	LINCOSAMIDE

Table S4: List of virulence pathways detected.

#OTU ID	KEGG_Description
K07344	type IV secretion system protein TrbL
K02418	flagellar protein FliO/FliZ
K02419	flagellar biosynthetic protein FliP
K02412	flagellum-specific ATP synthase [EC:3.6.3.14]
K02413	flagellar FliJ protein
K02410	flagellar motor switch protein FliG
K02411	flagellar assembly protein FliH
K02416	flagellar motor switch protein FliM
K02417	flagellar motor switch protein FliN/FliY
K02414	flagellar hook-length control protein FliK
K02415	flagellar FliL protein
K03230	type III secretion protein SctV
K03219	type III secretion protein SctC
K06604	flagellar rod protein FlaI
K02399	flagella synthesis protein FlgN
K02395	flagellar protein FlgJ
K02394	flagellar P-ring protein precursor FlgI
K02397	flagellar hook-associated protein 3 FlgL
K02396	flagellar hook-associated protein 1 FlgK
K02391	flagellar basal-body rod protein FlgF
K02390	flagellar hook protein FlgE
K02393	flagellar L-ring protein precursor FlgH
K02392	flagellar basal-body rod protein FlgG
K11898	type VI secretion system protein ImpE
K11899	type VI secretion system protein ImpD
K11896	type VI secretion system protein ImpG
K11897	type VI secretion system protein ImpF
K11894	type VI secretion system protein ImpI
K11895	type VI secretion system protein ImpH
K11892	type VI secretion system protein ImpK
K11893	type VI secretion system protein ImpJ
K11890	type VI secretion system protein ImpM
K11891	type VI secretion system protein ImpL
K04058	type III secretion protein SctW
K04059	type III secretion protein SctX
K04050	type III secretion protein SctE
K04051	type III secretion protein SctG
K04052	type III secretion protein SctH
K04053	type III secretion protein SctI
K04054	type III secretion protein SctK
K04056	type III secretion protein SctO
K04057	type III secretion protein SctP
K02404	flagellar biosynthesis protein FlhF
K13626	flagellar assembly factor FliW
K11931	biofilm PGA synthesis lipoprotein PgaB [EC:3.-.-.-]

K11935	biofilm PGA synthesis protein PgaA
K11937	biofilm PGA synthesis protein PgaD
K11936	biofilm PGA synthesis N-glycosyltransferase PgaC [EC:2.4.-.-]
K11919	type VI secretion system lysozyme-related protein
K11918	type VI secretion system protein
K11913	type VI secretion system protein
K11911	type VI secretion system protein VasL
K11910	type VI secretion system protein VasJ
K03228	type III secretion protein SctT
K03229	type III secretion protein SctU
K03222	type III secretion protein SctJ
K03223	type III secretion protein SctL
K03220	type III secretion protein SctD
K03221	type III secretion protein SctF
K03226	type III secretion protein SctR
K03227	type III secretion protein SctS
K03225	type III secretion protein SctQ
K02407	flagellar hook-associated protein 2
K02406	flagellin
K12148	biofilm regulator BssS
K02665	type IV pilus assembly protein PilP
K02664	type IV pilus assembly protein PilO
K02666	type IV pilus assembly protein PilQ
K02661	type IV pilus assembly protein PilK
K02663	type IV pilus assembly protein PilN
K02662	type IV pilus assembly protein PilM
K02409	flagellar M-ring protein FliF
K02408	flagellar hook-basal body complex protein FliE
K02401	flagellar biosynthetic protein FlhB
K02400	flagellar biosynthesis protein FlhA
K02403	flagellar transcriptional activator FlhD
K02402	flagellar transcriptional activator FlhC
K03204	type IV secretion system protein VirB9
K03205	type IV secretion system protein VirD4
K03200	type IV secretion system protein VirB5
K03201	type IV secretion system protein VirB6
K03202	type IV secretion system protein VirB7
K03203	type IV secretion system protein VirB8
K02422	flagellar protein FliS
K02421	flagellar biosynthetic protein FliR
K02388	flagellar basal-body rod protein FlgC
K02389	flagellar basal-body rod modification protein FlgD
K02386	flagella basal body P-ring formation protein FlgA
K02387	flagellar basal-body rod protein FlgB
K02385	flagellar protein FlbD

K06603	flagellar protein FlaG
K06602	flagellar protein FlaF
K06601	flagellar protein FlbT
K04060	type III secretion protein SctY
K04061	flagellar biosynthesis protein
K02423	flagellar protein FliT
K02420	flagellar biosynthetic protein FliQ
K04049	type III secretion protein SctB
K04562	flagellar biosynthesis protein FlhG
K11909	type VI secretion system protein VasI
K11904	type VI secretion system secreted protein VgrG
K11905	type VI secretion system protein
K11906	type VI secretion system protein VasD
K11907	type VI secretion system protein VasG
K11900	type VI secretion system protein ImpC
K11901	type VI secretion system protein ImpB
K11902	type VI secretion system protein ImpA
K11903	type VI secretion system secreted protein Hcp
K03196	type IV secretion system protein VirB11
K03198	type IV secretion system protein VirB3
K03199	type IV secretion system protein VirB4
K03194	type IV secretion system protein VirB1
K03195	type IV secretion system protein VirB10
K03197	type IV secretion system protein VirB2
